# Metabolism, Energetics, and Lipid Biology in the Podocyte – Cellular Cholesterol-Mediated Glomerular Injury

**DOI:** 10.3389/fendo.2014.00169

**Published:** 2014-10-14

**Authors:** Sandra Merscher, Christopher E. Pedigo, Armando J. Mendez

**Affiliations:** ^1^Peggy and Harold Katz Family Drug Discovery Center and Division of Nephrology and Hypertension, Department of Medicine, University of Miami, Miami, FL, USA; ^2^Division of Diabetes, Endocrinology and Metabolism, Department of Medicine, Diabetes Research Institute, University of Miami, Miami, FL, USA

**Keywords:** cholesterol, podocyte, renal disease, kidney disease, glomerular disease, ABCA1, reverse cholesterol transport, apolipoprotein

## Abstract

Chronic kidney disease (CKD) is associated with a high risk of death. Dyslipidemia is commonly observed in patients with CKD and is accompanied by a decrease in plasma high-density lipoprotein, and an increase in plasma triglyceride-rich lipoproteins and oxidized lipids. The observation that statins may decrease albuminuria but do not stop the progression of CKD indicates that pathways other than the cholesterol synthesis contribute to cholesterol accumulation in the kidneys of patients with CKD. Recently, it has become clear that increased lipid influx and impaired reverse cholesterol transport can promote glomerulosclerosis, and tubulointerstitial damage. Lipid-rafts are cholesterol-rich membrane domains with important functions in regulating membrane fluidity, membrane protein trafficking, and in the assembly of signaling molecules. In podocytes, which are specialized cells of the glomerulus, they contribute to the spatial organization of the slit diaphragm (SD) under physiological and pathological conditions. The discovery that podocyte-specific proteins such as podocin can bind and recruit cholesterol contributing to the formation of the SD underlines the importance of cholesterol homeostasis in podocytes and suggests cholesterol as an important regulator in the development of proteinuric kidney disease. Cellular cholesterol accumulation due to increased synthesis, influx, or decreased efflux is an emerging concept in podocyte biology. This review will focus on the role of cellular cholesterol accumulation in the pathogenesis of kidney diseases with a focus on glomerular diseases.

The glomerulus is a highly specialized structure that ensures that essential proteins are retained in the blood through the selective ultrafiltration of plasma (1). Intensive research has outlined the crucial role of podocytes and the slit diaphragm (SD) in the size-selective filtration of proteins from plasma. Podocytes are visceral glomerular epithelial cells consisting of a cell body, major and minor foot processes. Numerous foot processes from neighboring podocytes form a unique interdigitating pattern leaving filtration slits between them, which are bridged by a 40-nm wide SD (2, 3). The integrity of this filtration barrier is important to prevent the loss of protein into the urine (proteinuria). Research of the past two decades has revealed that the SD is a lipid raft-like structure that contains multi-protein complexes, including ion channels, receptors (transcient receptor potential cation channel 6, TRPC6), transmembrane proteins (nephrin, NPHS1), integral membrane proteins (podocin, NPHS2), structural proteins (alpha-actinin-4, ACTN4), signaling adaptors (CD2 associated protein, CD2AP), and other proteins involved in cell signaling, and that proper signaling at the SD is essential for proper glomerular filtration. Thus, mutations in genes coding for SD proteins were shown to cause proteinuria-associated nephropathies (4–8). The observation that the SD represents a lipid-raft like structure suggested that cholesterol might play an important role in granting proper localization of SD proteins and initiated recent research to investigate the role of cellular cholesterol metabolism in proteinuric kidney disease. It was shown that binding of SD proteins such as podocin to cholesterol is necessary for the podocin-dependent activation of TRPC6 (9, 10), further demonstrating the importance of cholesterol in the proper function and localization of SD proteins. This review will focus on the role of cellular cholesterol imbalance in the pathogenesis of proteinuric glomerular diseases.

## Cellular Cholesterol Homeostasis

**Figure 1:**
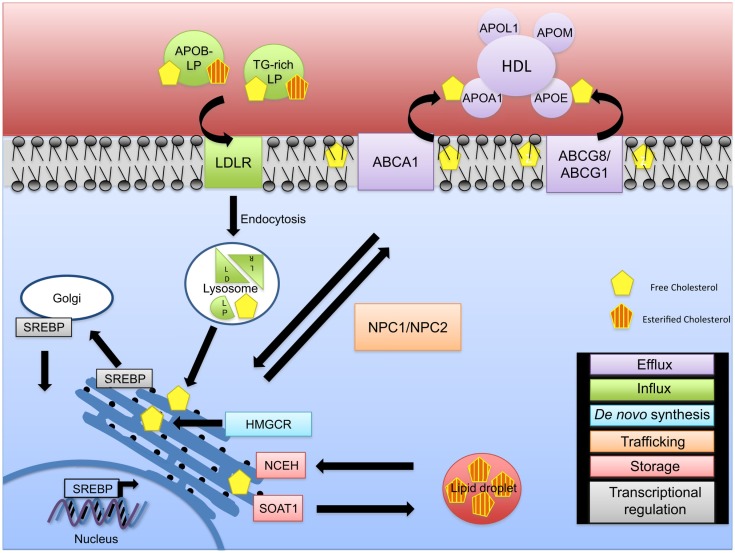
**Intracellular cholesterol trafficking**. The fine regulation of cholesterol homeostasis is maintained via different mechanisms. *De novo* synthesis (blue) of free cholesterol occurs via the rate-limiting enzyme HMG-CoA reductase (HMGCR) at the endoplasmic reticulum (ER). In the event of a cellular cholesterol deficit, cholesterol influx (green) mediated through APOB-rich lipoproteins and triglyceride-rich (TG-rich) lipoproteins occurs. LDLR/lipoprotein complexes are internalized via endocytosis and transported to the lysosome for degradation resulting in LDL and VLDL remnants, thus releasing free cholesterol. As excess free cholesterol is toxic, it is transported to the plasma membrane via NPC1/2 for efflux by an ABCA1-ApoAI/L1- or ABCG1/8-HDL-mediated mechanism (purple), or it is converted to cholesteryl esters via SOAT1 leading to the formation of cholesterol enriched lipid droplets (red). Cholesteryl esters can be converted back to unesterified (free) cholesterol via NCEH. Cholesterol pathways are regulated on a transcriptional level (gray). During cholesterol deficits, SREBP is transported to the Golgi apparatus and cleaved, allowing its translocation to the nucleus to regulate expression of cholesterol genes.

Cellular cholesterol homeostasis is regulated by *de novo* synthesis, cholesterol influx, and efflux (11) and is crucial for proper cell function (Figure 1). Cholesterol synthesis is tightly regulated through the action of the sterol regulatory element binding proteins (SREBPs) that play a critical role in the regulation of genes involved in cholesterol and fatty acid synthesis and of the low-density lipoprotein receptor (LDLR) gene. Of the three known isoforms of SREBP (SREBP-1a, SREBP-1c, and SREBP-2), SREBP-2 preferentially activates genes involved in cholesterol metabolism, whereas SREB-1c primarily acts on genes important in fatty acid and triglyceride metabolism (12, 13). SREBPs reside in the endoplasmatic reticulum (ER) membrane bound in complexes with SREBP cleavage-activating protein (SCAP) (14–16). When cellular cholesterol levels are low in the ER membrane, a conformational change occurs in SCAP, allowing SREBPs to be transported to the Golgi apparatus where two sequential proteolytic cleavages occur allowing for the release of the active SREBP fragment that can transcriptionally regulate several sterol response element genes including 3-hydroxy-3-methyl-glutaryl-CoA reductase (HMG-CoA reductase, HMGCR), the rate-limiting enzyme of cholesterol biosynthesis. When ER membrane cholesterol levels are elevated, SCAP remains associated with SREBP and prevents its transport and activation in the Golgi. This pathway provides a feedback mechanism to prevent the over accumulation of cellular cholesterol (Figure 1).

In the blood, cholesterol is transported by two major lipoproteins, low-density lipoprotein (LDL) and high-density lipoprotein (HDL). Circulating LDL is the major source for cholesterol uptake into cells in a LDLR dependent manner (17). Binding of cholesterol-containing LDL to its receptor is followed by endocytosis and fusion of the LDL-containing vesicle with a lysosome. Lysosomal enzymes digest the LDL, breaking down the proteins to amino acids and liberating the free cholesterol. A concerted action between Niemann–Pick C1 and C2 proteins (NPC1 and NPC2) allows for the transport of unesterified (free) cholesterol from the endosome/lysosome complex to the ER and then to the plasma membrane (18, 19). The increase in cellular cholesterol induces cellular cholesteryl ester formation through activation of sterol-O-acyltransferase 1 (SOAT1 or acyl-Coenzyme A: cholesterol acyltransferase, ACAT1) and reduces cholesterol synthesis through inhibition of HMGCR and decreases LDLR synthesis (17). Abnormal accumulation of free cholesterol leads to cell toxicity underlining the importance of cholesterol esterification in maintaining proper levels of free cholesterol needed for optimal cell and cell membrane function (20–22). Enzymes involved in conversion of free cholesterol to cholesteryl esters and *vice versa* at the ER are SOAT1 and neutral cholesterol ester hydrolase 1 (NCEH) (23). Cellular levels of cholesterol are also modulated through the reverse cholesterol transport pathways that promote cholesterol efflux to HDL (Figure 1).

Efflux of cellular cholesterol occurs through several distinct pathways (24). Cholesterol efflux by aqueous diffusion is a bi-directional, energy independent process and involves equilibrium of cholesterol molecules between cellular membranes and any acceptor, including HDL. Scavenger receptor B-I (SR-BI) mediates the selective uptake of HDL cholesteryl esters into cells, and also facilitates the passive efflux of cholesterol from cells to HDL. Third, efflux of phospholipids and cholesterol mediated by the ATP-binding cassette transporter A1 (ABCA1) transporter is an active, energy-requiring process that requires the presence of extracellular lipid-poor apolipoproteins (APO proteins) including APOAI and APOE. Another ATP-binding cassette transporter, ABCG1, enhances efflux by increasing desorption of plasma membrane cholesterol and enhancing removal by HDL (Figure 1). The contribution of the various efflux pathways to reverse cholesterol transport, especially *in vivo*, remains unknown and it is likely that all pathways contribute to the overall extent of reverse cholesterol transport. While the first step in reverse cholesterol transport is removal of cellular cholesterol to HDL, several other key steps in reverse cholesterol transport involve cholesterol esterification by lecithin-cholesterol acyltransferase (LCAT) and transfer of HDL-associated cholesteryl esters to APOB containing lipoproteins through the action of cholesteryl ester transferase protein (CETP) and subsequent uptake of cholesterol by the liver for excretion or redistribution.

Dyslipidemia is a common disorder observed in subjects with chronic kidney disease (CKD). The most common lipid abnormalities observed are elevated fasting triglyceride levels and reduced HDL cholesterol, while total cholesterol and LDL cholesterol can be elevated or in the normal range and is often below normal in subjects with renal failure (25–27). In addition to the quantitative changes in lipoprotein levels, there are a multitude of qualitative changes in the lipoproteins of CKD patients that include reduced clearance rates of triglyceride-rich lipoproteins resulting in increased blood levels of atherogenic remnant particles (28), increased levels of small dense LDL particles (29), increased levels of oxidized LDL (30), altered HDL subfraction distribution with reductions in larger HDL particles consistent with delayed maturation of HDL (31), reduced LCAT activity (32), and decreased HDL anti-oxidant and anti-inflammatory activity (27). Each of these lipoprotein abnormalities can contribute to the increased risk of cardiovascular disease observed in patients with CKD and likely contribute to altered cellular cholesterol homeostasis in kidney cells.

Defective cellular cholesterol trafficking was described in diseases of genetic and non-genetic origin with and without renal involvement (33–36). Hypercholesterolemia due to impaired reverse cholesterol transport and abnormalities of lipid metabolism are common features observed in patients with end-stage renal disease (ESRD), including patients on hemodialysis, patients with diabetic kidney disease (DKD), nephrotic syndrome, and uremia (37–40). In addition to hypercholesterolemia in the blood, lipid accumulation in glomeruli of patients with kidney disease was described. Although hyperlipidemia is a known risk factor for the development of cardiovascular disease and atherosclerosis, the role of hyperlipidemia as a cause for the development of renal diseases and the mechanisms leading to glomerular lipid accumulation remain less understood (41–44).

Within the kidney, the glomerulus and proximal tubules express various apolipoproteins and proteins important in lipid and cholesterol metabolism. More than 30 years ago, apoA1 and apoB were the first apolipoproteins shown to be synthesized in kidneys from chickens suggesting that the kidney might be a major source for the synthesis of plasma lipoproteins (45, 46). Later, it was shown that both are secreted as constituents of lipoprotein particles from kidney cells (47). APOM, a lipoprotein associated with HDL (48), APOE (49, 50), APOB (50), and APOL1 (51), was consequently shown to be expressed in human kidneys, and we recently demonstrated that human glomerular podocytes express ABCA1, HMGCR, and LDL receptor (52). These observations indicate the possibility of apolipoprotein-mediated cholesterol homeostasis in kidney cells and may suggest that dysregulated cholesterol homeostasis may represent a pathogenic mechanism in kidney disease.

## Defective Cellular Cholesterol Trafficking in Glomerular Diseases of Genetic Origin

### APOE in glomerular disease

Within the glomerulus, podocytes are able to uptake LDL, APOB, and APOE containing lipoproteins. Their capacity to uptake APOB and APOE containing lipoproteins is increased when compared to LDL, and this process is associated with the suppression of cellular sterol synthesis and cholesteryl ester formation (50).

Lipoprotein glomerulopathy [LPG, Online Mendelian Inheritance in Man (OMIM) #611771] is a rare genetic disorder mainly affecting people of Japanese and Chinese origin that is caused by a heterozygous mutation in the *APOE* gene on chromosome 19q13. Several mutations in *APOE* were identified resulting in the expression of a dysfunctional APOE protein with impaired LDL receptor binding (34, 53, 54). Patients with LPG have proteinuria and nephrotic syndrome with elevated serum APOE levels, abnormal lipoprotein deposition in glomerular capillaries in form of laminated thrombi that contain APOB and APOE, a variable degree of mesangial proliferation, and dysbetalipoproteinemia (55, 56). Occasionally, a similar renal phenotype can be observed in type III hyperlipoproteinemia, a disease that is characterized by homozygous mutation in the ε2 allele of the *APOE2* gene. The kidney phenotype includes proteinuria, glomerulosclerosis with mesangial and interstitial foam-cell accumulation, and the presence of intraglomerular thrombi (57, 58). Genetic polymorphisms in *APOE* were shown to differentially affect LDLR binding. Thus, the APOE2 variant binds poorly to LDLR, whereas the APOE4 variant binds with high affinity (59). The *APOE2* variant was associated with persistent proteinuria and renal failure in patients with non-insulin-dependent diabetes mellitus (NIDDM) (60) and may contribute to the severity of the renal phenotype in IgA nephropathy (61). Conversely, the polymorphism in *APOE4* is thought to constitute a protective factor in patients with NIDDM (62).

Constitutive *ApoE*^−/−^ mice generated through partial replacement of exon 3 and part of intron 3 of the *ApoE* gene by a neomycin cassette (63) are a model for experimental atherosclerosis and develop a renal phenotype similar to that observed in patients with type III hyperlipoproteinemia and LPG (64–66). Kidneys from *ApoE*^−/−^ mice showed glomerular macrophage infiltration with foam-cell formation, deposition of extracellular matrix, glomerular hyperplasia, mesangiolysis, and lipid deposits in glomerular capillaries at 24 weeks, which further progressed to a LPG-like phenotype at 36 weeks (66). In a different study, in which the *APOE*-Sendai mutation (Arg145Pro mutation found in patients with LPG) was introduced into *ApoE*^−/−^ mice showed that, although similar, the LPG-like lesions observed in the *APOE*-Sendai mice were different from the lesions in aged *ApoE*^−/−^ mice (65, 67) indicating that *APOE* variants may contribute to a differential phenotype in LPG. *ApoE*^−/−^ mice, which were fed a high fat diet developed a renal phenotype at 16 weeks of age with lipid droplet and cholesterol crystal accumulation within the glomerulus. This phenotype was associated with decreased basal plasma renin concentrations and low mean arterial blood pressure, indicating impaired renin-angiotensin system (RAS) function. After renal artery constriction, these mice were unable to increase renin secretion from the juxtaglomerular cells, demonstrating a lack of a normal response to renal hypoperfusion (68). Similar glomerular changes were observed in another study when male mice were fed a high cholesterol diet for 20 weeks (69) indicating that high fat diet accelerates the glomerular injury phenotype. Likewise, streptozotocin (STZ) injected into *ApoE*^−/−^ mice accelerated renal injury when compared to non-diabetic mice and was attenuated when advanced glycation end product (AGE) accumulation was prevented by the use of an AGE formation inhibitor. Renal changes observed in diabetic *ApoE*^−/−^ mice included increased albuminuria and structural changes in glomeruli and tubulointerstitium (70). Taken together, these studies suggest that ApoE deficiency and mutations that affect ApoE binding to LDLR and the resulting hyperlipidemia play a role in rendering renal cells more susceptible to glomerular injury.

In further support of this hypothesis, mice with a combined deficiency of inhibitor of differentiation 3 (*Id3*) and *ApoE*^−/−^ spontaneously develop glomerulonephritis characterized by lipid deposition specifically in glomeruli, the presence of enlarged hypercellular glomeruli, mesangial expansion, and increased extracellular matrix deposition (71). *ApoE* deficiency also accelerates the renal phenotype in MRL-*Fas^lpr^* mice, a mouse model of human lupus (72). Further, decreased ApoE expression in glomerular podocytes is observed in Tg26 (human immunodeficiency virus, HIV) and Nef (negative regulatory factor) transgenic mice, two models of HIV-associated nephropathy (HIVAN) (73), whereas increased ApoE and ApoB expression was found in association with accumulation of oil red O positive lipid droplets in glomerular visceral epithelial cells and mesangial cells in rats with puromycin aminonucleoside or adriamycin induced nephrosis (74). Increased glomerular APOE expression was observed in patients with idiopathic nephrotic syndrome but it is rather a rare occurrence in focal segmental glomerulosclerosis (FSGS) (75).

### APOM in glomerular disease

APOM is a 26-kDa apolipoprotein and is a member of the lipocalin family expressed in the liver and in the kidney. In the plasma, APOM is associated with HDL particles (48); in kidney proximal tubular cells, APOM binds to megalin, thus preventing its excretion in the urine by megalin-mediated endocytosis (76). Mutations, polymorphisms, or allelic variants of the APOM gene have not been associated with any glomerular phenotype. Although APOM is strongly expressed in kidney tubular epithelial cells, it is not found in the urine of mice or human beings. However, megalin knockout mice were shown to be characterized by urinary loss of low molecular weight proteins underlining an important role of proximal tubules in resorption of proteins (77). Megalin is expressed in glomerular podocytes where it can bind to α-galactosidase A, suggesting a potential role in Fabry disease, and it may act as a pathogenic antigen in membranous glomerulonephritis (78, 79).

### APOL1 in glomerular disease

APOL1 is a secreted HDL-associated protein that binds to APOA1 and promotes cholesterol efflux. It is coded by a gene on human chromosome 22q12, and *APOL1* mutations are associated with increased susceptibility to FSGS (OMIM #612551), HIVAN, and hypertensive nephropathy in patients of African ancestry (80–82). Using immunofluorescence staining of normal human kidneys, APOL1 was localized to podocytes of the glomerulus, the promixal tubules, and the extraglomerular arterial endothelium. In kidney biopsies from patients with FSGS and HIVAN, APOL1 expression levels in podocytes were found decreased and *de novo* appearance of APOL1 within cells of the arterial medial wall was observed (51). The observation that normal human podocytes express APOL1 opens up the possibility that APOL1 may be contributing to cellular cholesterol homeostasis under physiological conditions. Likewise, it is possible that decreased podocyte APOL1 expression under disease conditions leads to decreased cholesterol efflux from podocytes and cellular cholesterol accumulation, thus contributing to the pathogenesis of glomerular diseases such as FSGS and HIVAN. Future studies to investigate the role of the different APOL1 variants and their role in podocyte cholesterol homeostasis under physiological and disease conditions are needed to shed light on their contribution to the pathogenesis of glomerular diseases.

### LCAT in glomerular disease

Familial *LCAT* deficiency (OMIM #245900) is a rare genetic disorder caused by a mutation in the *LCAT* gene. LCAT is a HDL-associated enzyme that converts cholesterol to cholesteryl esters by transfer of the SN2 fatty acid from a phosphatidylcholines (lecithins) with the formation of lyso-phosphatidylcholine on the surface of HDLs. It, therefore, plays an important role in reverse cholesterol transport from peripheral tissues to the liver. *LCAT* deficiency leads to cholesterol accumulation in many tissues (83, 84). Patients with familial *LCAT* deficiency are characterized by diffuse corneal opacities, target cell hemolytic anemia, and proteinuria with chronic progressive glomerulopathy resulting in renal failure. Massive lipid deposits in the glomerular basement membrane (GBM) and in the mesangial region can be detected (85, 86). The appearance of abnormal choleastic lipoprotein, LpX, in the plasma of the patients was described (84, 87, 88).

Lecithin-cholesterol acyltransferase was originally shown to be expressed only in liver (89) but later also in brain, testis, ileum, kidney, spleen, and adrenal tissue (84, 90). More detailed analysis of LCAT expression in kidneys from Mongolian gerbils by immunohistochemistry showed expression mainly in the proximal and distal convoluted tubules and in the collecting duct epithelial cells (91).

*Lcat* deficient mice are characterized by reduced total cholesterol, HDL cholesterol, and APOA1 plasma levels (92, 93). When fed a regular chow diet, *Lcat* deficient mice show no glomerular phenotype. However, when fed a high fat diet, *Lcat* deficient mice developed glomerular lesions including reduction of vascular space, mesangial expansion and sclerosis, and increased extracellular matrix with accumulation of lipid droplets and macrophages. Lipid droplets in glomeruli were composed of free cholesterol and polar lipids as shown by filipin and oil red O staining. Interestingly, the glomerular phenotype was only observed in mice that simultaneously accumulated LpX (94). In support of the observation that elevated plasma LpX levels are necessary to mediate the glomerular phenotype, double mutant mice with *Lcat* deficiency that constitutively overexpress a NH2-terminus segment of SREBP-1a, which leads to accumulation of predominantly LpX in plasma were shown to spontaneously develop glomerular lesions with glomerular and tubulointerstitial lipid deposits at 6 months (95).

### NPC1 and NPC2 in glomerular disease

Niemann–Pick disease type C (OMIM#607616) is a genetic, neurogenerative disorder caused by mutations in the genes *NPC1* on chromosome 18q11 in the vast majority of the cases, and *NPC2* on chromosome 14q24. Patients present with ataxia, vertical supranuclear gaze palsy (VSGP), and dementia. Mutations in this gene(s) lead to the inability to transport free cholesterol between intracellular compartments. As a consequence, free cholesterol and other lipids accumulate in late endosomes/lysosomes in all organs including the kidney simultaneously causing a delayed response in sterol-responsive pathways to exogenous cholesterol (96, 97). NPC1 and NPC2 are expressed in the kidney (98, 99). NPC2 expression was further localized to the distal and proximal convoluted tubules of the kidney (99). Although rather rare, Niemann–Pick disease-associated renal pathology was described in biopsies from patients and included foamy podocytes, vacuolated tubular epithelial cells, and collections of foam cells in the interstitium (100). Association with a phenotype resembling membranoproliferative glomerulonephritis type II was described as well (101). These observations indicate that cholesterol accumulation in glomerular or tubular cells contributes to the renal pathogenesis observed in Niemann–Pick disease. In *Npc1* deficient mice, a single injection with hydroxy-propyl-beta cyclodextrin, a cholesterol-depleting agent, was shown to increase the life span and weekly injections normalized cholesterol metabolism in nearly every organ (102–104).

### ABCA1 in glomerular disease

Tangier disease (OMIM #205400) is an autosomal recessive and familial hypoalphalipoproteinemia (FHA, OMIM #604091), an autosomal dominant disorder caused by a mutation in the *ABCA1* gene on chromosome 9q31 leading to accumulation of esterified cholesterol in tissues due to impaired reverse cholesterol transport resulting in reduced levels of plasma HDL (105–108). Patients present with heterogeneous clinical symptoms including liver, spleen, lymph node, and tonsil enlargement and peripheral neuropathy in children and adolescents. Occasionally, cardiovascular disease is observed in adults but the presence of a renal phenotype in Tangier patients is extremely rare (109, 110). FHA is the more common disease and is, like Tangier disease, characterized by low plasma HDL but without the clinical manifestations of Tangier disease. *Abca1* deficient mice as a model for Tangier disease were generated by partial (111) or complete (112) replacement of the exons encoding the first ATP-binding cassette of *Abca1*. When fed on a high fat diet, *Abca1* deficient mice were characterized by significantly decreased plasma HDL levels and lipid accumulation in several tissues including the kidney. Phenotypical differences were observed within the two models of *Abca1* deficiency, possibly due to differences in the high fat diet composition or to slight differences in the targeting strategies (111, 112). In the study by Christiansen-Weber, a glomerular phenotype was observed, which included the presence of a thickened and “split” GBM and mesangial cell proliferation. Furthermore, immunoglobulin and C3 complement complexes characteristic of menbranoproliferative glomerulonephritis were detected (111), whereas no renal phenotype was described in the other study. The observation that *Abca1* deficient mice develop a glomerular phenotype on a high fat diet deficiency may indicate that *Abca1* deficiency leads to increased susceptibility for the development of renal disease.

## Defective Cellular Cholesterol Trafficking in Glomerular Diseases of Non-Genetic Origin

### Defective renal cholesterol homeostasis in diabetic kidney disease

Lipid droplets in kidney biopsies from patients with DKD were first identified by Kimmelstiel and Wilson (42) and were more recently localized within podocyte foot processes of patients with DKD (41). We recently demonstrated that glomerular *ABCA1* expression is decreased in patients with type 2 diabetes (T2D) and early DKD and in human podocytes treated with the sera from patients with type 1 diabetes (T1D) and DKD in the absence of changes in *LDLR* and *HMGCR* expression (52). These observations indicate that lipid accumulation in podocytes due to defective cellular cholesterol homeostasis may play an important role in glomerular injury in DKD.

In support of these observations, lipid droplets in various experimental models of DKD have been reported. In two models for DKD with type 1 diabetes, Akita, and OVE26 mice, renal triglycerides and cholesterol accumulation was described and was associated with increased renal cholesterol synthesis and decreased efflux. This coincided with increased expression of SREBP-2 and HMGCR and decreased expression of liver X receptor (LXR)-α, LXR-β, and ABCA1 in kidneys of these mice (113). In diabetic STZ-injected DBA2/J mice, the DKD phenotype, which includes albuminuria, mesangial expansion, fibrosis and lipid accumulation in glomeruli, and tubulointerstitium is associated with increased HMGCR expression in kidneys. Aliskiren (a renin inhibitor) and valsartan (an angiotensin II receptor antagonist) reduced albuminuria and glomerulosclerosis in these animals and was associated with decreased HMGCR expression and decreased lipid accumulation in glomeruli (114), suggesting a potential crosstalk between the RAS and lipid metabolism in the kidney. FvB-Lepr*^db/db^* mice, a model for DKD with type 2 diabetes, spontaneously develop a renal DKD-like phenotype that includes glomerulosclerosis, tubulointerstitial fibrosis, GBM thickening, and proteinuria. In addition, renal triglyceride and cholesterol accumulation in glomeruli and tubules associated with increased activity of SREBP-1 and -2 was observed (115). Likewise, renal cholesterol accumulation in high fat/sucrose-fed (116) (type 2 diabetes model for DKD) and STZ-induced rats (type 1 diabetes model for DKD) was associated with increased expression of SCAP, SREBP-2, HMGCR, and LDLR. Atorvastatin, a lipid-lowering agent with antioxidative and anti-inflammatory effects reduced cholesterol synthesis in the kidneys improving renal function and kidney morphology in these models (116).

It should be noted that STZ has also been shown to impair renal function during the first week after injection in rodents affecting not only proteinuria but also renal microcirculation and oxidative pathways (117, 118). Studies to elucidate the contribution of hyperglycemia after STZ treatment or STZ-induced renal toxicity demonstrated that a majority of the renal damage was a result of the diabetic condition and only early proteinuria was caused by the nephrotoxic effects of STZ (119).

In the non-obese diabetic (NOD) mouse model of T1D, ABCA1 protein expression was decreased in kidneys and circulating macrophages, and associated with increased cholesterol content indicating that impaired reverse cholesterol transport may contribute to kidney phenotype observed in diabetic NOD mice (120). Diabetes-induced reduction of kidney ABCA1 expression in association with cholesterol accumulation was observed in cyclophosphamide-induced diabetic NOD and in LDLR-deficient-GP (glycoprotein) lymphocytic choriomeningitis virus (LCMV)-induced diabetic mice (121). In BTBR *ob/ob* mice, a type 2 diabetes model of DKD, cholesterol accumulation occurs in kidneys, a phenotype that can be prevented by cholesterol depletion with cyclodextrin, underlining the causative role of cholesterol accumulation in glomerular injury (52). Together these studies indicate that cholesterol accumulation due to impaired reverse cholesterol transport occurs in glomerular podocytes and contribute to the pathogenesis of DKD. As statins, which decrease cholesterol synthesis do not substantially affect the progression of kidney disease (122–124), other specific strategies that facilitate transport of sequestered cholesterol such as targeting reverse cholesterol transport are needed and may prove beneficial in treatment of DKD.

### Defective renal cholesterol homeostasis in non-diabetic kidney disease

Chronic renal failure induced by 5/6 nephrectomy in rats is associated with neutral lipid accumulation in the remnant renal tissue, glomerulosclerosis and tubulointerstitial injury, and proteinuria. In remnant kidneys, increased expression of LXR, ABCA1, ABCG1, ACAT1, SR-B1, scavenger receptor A1 (SR-A1), lectin-type oxidized LDL receptor (LOX-1), carbohydrate-responsive element binding protein (ChREBP), fatty acid synthase (FAS), and acyl-CoA carboxylase (ACC) was detected, whereas expression of SREBP-1, SREBP-2, HMGCR, peroxisome proliferator-activated receptor alpha (PPAR-α), fatty acid binding protein (L-FABP), and carnitine palmitoyltransferase 1A (CPT1A) was decreased (125, 126). Cholesterol accumulation in proximal tubules is associated with HMGCR activation in acute kidney injury (AKI) (127, 128). The observation that cholesterol accumulation can result from glomerular injury suggested that renal cholesterol accumulation may represent a mechanism of stress response (129). In a model of experimental glomerulonephritis with concomitant proteinuria, cholesterol accumulation occurred in the renal cortex, i.e., the glomerulus and proximal tubular cells. While esterified cholesterol accumulation was present in glomerular and tubular cells, free cholesterol accumulation occurred in proximal tubular cells and was associated with increased ABCA1 and decreased SR-B1 expression (130).

Hypoxia-induced dyslipidemia is a phenomenon that was first described decades ago (131–133) and shown to play an important role in foam-cell formation and cytokine secretion in atherosclerosis and in non-alcoholic steatohepatitis (NASH) (134–137). Hypoxia is also a pathological feature observed in kidney disease and hypoxia-inducible factors (HIFs), such as HIF-1 and HIF-2 are thought to play an important role in AKI. HIFs initiate the transcription of a variety of genes involved in the regulation of tissue oxygen levels, glucose metabolism, apoptosis, lipid metabolism, and immune responses (138–143). HIF target genes include hemeoxygenase-1, vascular endothelial growth factor, plasminogen activator inhibitor-1, tissue-inhibitor of metalloproteinase-1, and connective tissue growth factor (139, 142, 144), all of which have been linked to the pathogenesis of a variety of glomerular diseases including DKD, FSGS, and HIVAN (145, 146). HIF-1 was shown to contribute to lipid accumulation by increasing lipid influx and synthesis in hepatocytes through increased LDL and very low-density lipoprotein (VLDL) uptake and increased levels and activity of HMGCR, respectively (147). More recently, it was demonstrated that activation of HIF-2 can also induce changes in hepatic lipid metabolism leading to the development of severe fatty liver disease in mice (148). Additionally, hypoxia-inducible protein 2 (HIG2) was identified as a novel lipid droplet protein and as a specific target gene of HIF-1 (149), further underlining a role for hypoxia in altered lipid metabolism in disease. In the kidney, HIF-1 was localized to the tubular epithelia, whereas HIF-2 was found predominantly in glomerular (podocytes), endothelial, and interstitial cells (150, 151). The induction of HIFs or inhibition of HIF degradation through hypoxia seems to have protective effects in AKI (152–154). Chronic hypoxia may also contribute to the pathogenesis of DKD (155) and genes induced by hypoxia can promote tubulointerstitial injury and renal fibrosis. Thus, novel therapeutic approaches targeting mechanisms of hypoxia-induced transcription may prove beneficial in targeting dyslipidemia in a variety of kidney diseases.

### Inflammation-induced cellular cholesterol accumulation in glomerular disease

Inflammation, foam-cell formation, and lipid accumulation are characteristic occurrences associated with glomerulosclerosis. In non-alcoholic fatty liver disease (NAFLD), a two hit hypothesis proposed that a first hit such as obesity, T2D, or the initial lipid accumulation in the liver sensitizes it to a second hit such oxidative stress and proinflammatory cytokines ultimately causing hepatocellular injury and liver inflammation (156). In hepatocytes, tumor necrosis factor alpha (TNFα) or interleukin-1 beta (IL-1β) mediated inflammatory stress significantly reduced intracellular cholesterol efflux by inhibiting PPAR, LXR, and ABCA1 expression and increased LDLR and SREBP-2 expression and suggesting that inflammatory stress may exacerbates progression of fatty liver in NAFLD (157). We recently described an important role of circulating factors in the pathogenesis of DKD and showed that treatment of human podocytes with the sera from patients with DKD leads to cholesterol accumulation (52). Therefore, it seems possible that circulating inflammatory factors, such as TNFα or IL-1β, contribute to the progression of DKD. In support of this observation, TNFα is a major predictor of DKD progression in T1D and T2D (158–160) and inflammatory cytokines, such as TNFα or IL-1β, were shown to modify cholesterol-mediated LDL receptor regulation in mesangial cells. These studies suggest that inflammatory cytokines contribute to lipid-mediated renal damage (161, 162). Furthermore, IL-1β was shown to promote intracellular cholesterol accumulation in human mesangial cells via downregulation of ABCA1 (163). Interestingly, IL-1β treatment of hepatic and mesangial cells also interrupted LDLR feedback regulation, thus causing statin resistance (164), which might explain why statins are not effective in preventing the progression of DKD. More recently, Nod-like receptor protein 3 (Nlrp3) knockout mice were shown to be protected against obesity-induced renal fibrosis and microalbuminuria which, in wildtype mice, was associated with cholesterol and lipid accumulation in kidneys and increased SREBP-2, LDLR, and SREBP-1c expression (165). Whether cytokines contribute to lipid accumulation observed in podocytes and the exact mechanisms that lead to cytokine-induced cholesterol accumulation remain to be investigated.

## Concluding Remarks

Emerging data strongly suggest a role of impaired cholesterol homeostasis within glomerular cells as an important mediator of CKD. This is supported by the observation that a variety of glomerular diseases of genetic and non-genetic origin in humans and targeted mutations in mice that affect lipid and lipoprotein metabolism are associated with impaired glomerular structure.

Podocytes express genes and proteins that modulate cellular cholesterol homeostasis, such as LDLR, ABC transporters, and apolipoproteins involved in maintaining cellular cholesterol levels. Recent studies demonstrated that the dysregulation of a single gene involved in cholesterol homeostasis is usually not sufficient to cause a glomerular phenotype *per se* but renders renal cells more susceptible to glomerular injury. Based on these observations, we propose that in the kidney, similar to what was proposed in NAFLD, a first insult such as obesity, T2D, or an initial lipid accumulation occurs, which then sensitizes the kidney to a second insult such as oxidative stress, proinflammatory cytokines, or other stimuli ultimately leading to renal damage. Further research is needed to determine what are the exact stimuli and mechanisms that lead to glomerular cholesterol accumulation and ultimately to impaired glomerular function. In addition, significant advances in recent years have led to the identification of new regulators of cellular cholesterol homeostasis. The observation that circulating factors such as cytokines and hypoxia-induced transcription factors, such as HIFs, can directly regulate genes important for cholesterol homeostasis in hepatocytes opens new avenues to investigate if they also contribute to lipid dysregulation observed within glomerular cells. Such research will not only increase our insights into the physiology of cellular cholesterol homeostasis and trafficking in glomerular cells but it will also help to identify new drug targets and strategies to treat a variety of glomerular diseases, which are characterized by cellular dyslipidemia.

## Conflict of Interest Statement

Sandra Merscher is the inventor on pending or issued patents aimed to diagnose or treat proteinuric renal diseases. She stands to gain royalties from their future commercialization. The other co-authors declare that the research was conducted in the absence of any commercial or financial relationships that could be construed as a potential conflict of interest.
